# Food Safety Assessment and Nutraceutical Outcomes of Dairy By-Products: Ovine Milk Whey as Wound Repair Enhancer on Injured Human Primary Gingival Fibroblasts

**DOI:** 10.3390/foods13050683

**Published:** 2024-02-23

**Authors:** Carlotta Ceniti, Anna Di Vito, Rosa Luisa Ambrosio, Aniello Anastasio, Jessica Bria, Domenico Britti, Emanuela Chiarella

**Affiliations:** 1 Department of Health Sciences, University “Magna Græcia” of Catanzaro, 88100 Catanzaro, Italy; ceniti@unicz.it; 2 Laboratory of Morphology and Tissue Cell Biology, Department of Experimental and Clinical Medicine, University “Magna Græcia” of Catanzaro, 88100 Catanzaro, Italy; divito@unicz.it (A.D.V.); jessica.bria@studenti.unicz.it (J.B.); 3 Department of Veterinary Medicine and Animal Production, University of Naples Federico II, 80137 Naples, Italy; rosaluisa.ambrosio@unina.it (R.L.A.); anastasi@unina.it (A.A.); 4 Interdepartmental Center Veterinary Service for Human and Animal Health (CISVetSUA), University “Magna Græcia” of Catanzaro, 88100 Catanzaro, Italy; 5 Laboratory of Molecular Haematopoiesis and Stem Cell Biology, Department of Experimental and Clinical Medicine, University “Magna Græcia” of Catanzaro, 88100 Catanzaro, Italy

**Keywords:** food safety, dairy-by-product, hazard, health claim, nutraceutical, human primary gingival fibroblasts, cytotoxicity, wound healing, CD44, COLI, regenerative therapy

## Abstract

The valorization of milk whey appears to be a promising strategy for managing by-products from dairy food industries, which incur demanding economic costs for treatment and/or disposal. Thanks to its numerous bioactive components, whey is expected to be increasingly incorporated into foods in the future. We investigated the safety of ovine milk whey through in vitro experiments on human primary gingival fibroblast (HGF-1) proliferation and wound healing. Fibroblasts play a crucial role in the repair processes from the late inflammatory phase until the final stages. Cells treated with varying concentrations of ovine whey (0.01%, 0.1%, 1%, and 10%) were able to close wounds more rapidly than vehicle-treated cells. Time- and dose-dependent responses were observed in cell populations exposed to ovine whey. Specifically, wounds treated with 0.1% and 10% milk whey showed better migratory capabilities compared to those treated with 0.01% and 1% milk whey after 24 and 48 h. In addition, ovine milk whey stimulates extracellular matrix deposition, as evidenced by the increasing levels of CD44 antigen density evaluated through FACS analysis, as well as COL1A1 expression measured both via RT-qPCR and immunofluorescence. This phenomenon was particularly evident at concentrations of 0.01% and 10%. Ensuring quality and safety has become a major concern for health authorities in the food industry. Our findings suggest that ovine milk whey is safe and possesses regenerative properties. It facilitates tissue re-establishment following exposure to environmental stress, particularly accelerating gingival wound closure.

## 1. Introduction

In the Mediterranean region, there has been increased attention towards the abundance of industrial food by-products due to their availability, cost-effectiveness, and their significant quantities of bioactive compounds [1]. The valorization of milk whey appears to be a promising strategy for managing by-products from the dairy food industry, which has become a global concern due to the challenging economic costs associated with its treatment and disposal. 

It is estimated that the dairy industry sector produces millions of tons of by-products [2], representing approximately the third highest category in terms of the monetary value of food wastage, accounting for 17–20% of the total value of food waste (United Nations Environment Programme, 2021) [3].

Dairy by-products fall into the broader category of Animal By-Products (ABP), which, as defined by European Council Regulation No. 1069/2009, encompass products of animal origin or those derived from processes involving animals. These are not intended for human consumption and comprise various categories, including slaughterhouse by-products (such as animal carcasses, blood, and bones) and waste generated by animal processing facilities (such as wastewater sludge from slaughterhouses) [4].

Milk or cheese whey, which has a yellow-green color, typically accounts for 85–95% of the milk’s volume. It retains about 55% of the milk’s nutrients and approximately 20% of its total protein content [5], whereas “scotta” is the exhausted whey, the final by-product of two consecutive cheese-making processes [6]. For an extended period, products derived from milk processing have traditionally been regarded as waste [7]. Strategies for handling this waste included using it as fertilizer or animal feed, converting it to biogas or ethanol, or discharging it into municipal sewage systems or soil [8,9]. The last option mentioned could potentially lead to environmental pollution because of the elevated levels of dissolved organic substances, such as proteins, fats, and lactose. Therefore, the pursuit of economically viable and environmentally suitable alternatives for utilizing whey protein is of utmost importance. 

Several authors have highlighted the potential application of agri-food by-products in the production of food items, emphasizing their possible implications for human health and sustainability [10,11,12,13,14]. According to recent research, agri-food by-products, including dairy by-products, can be incorporated into various nutritionally enhanced products to boost their bioactive profile, fiber content, and antioxidant capacity, all while maintaining a high level of sensory acceptability. Verardo and colleagues [15] have summarized the extraction of phospholipids from dairy by-products like milk whey, buttermilk, and butter serum, illustrating their potential applications in improving human health. It is well known that milk proteins currently serve as the primary source of various biologically active components with a range of physiological benefits, including antimicrobial activity [16,17]. Some researchers have measured the serum proteins, including lactoferrin, lactoperoxidase, serum albumin, α-lactalbumin, and whey β-lactoglobulin, [18]. Moreover, whey from ovine milk, with its unique protein characteristics, holds great potential for the development of specific dry whey products. Indeed, ovine whey protein concentrate boasts an abundance of β-lactoglobulin and better foam overrun, foam stability, and gel strength compared to those from cows and goats [19]. The expectation is for a growing utilization and application of ovine milk whey due to these advantageous properties.

In this context, the valorization of waste products could have a significant impact on the agro-industrial economy, reducing the burden of food losses and waste as well as discovering poor and cheap sources of bioactive compounds. These substances should be added to functionalized foods with the aim of improving their nutritional and nutraceutical profile. In this regard, it is necessary to demonstrate their safety when administered to consumers though foods. Ensuring quality and safety has emerged as the major priority for health authorities operating in the food industry. Food quality primarily encompasses attributes like nutritional value, taste, appearance, texture, and aroma, which must be preserved to ensure nutritional quality and consumer acceptability. However, it is important to acknowledge that exposure to certain foods or by-products can pose risks to human health and other living organisms [20,21]. As highlighted by Socas-Rodriguez (2021), food by-products require safety assessment for their use in humans or animals, and safety evaluations are scarce [22]. Therefore, due to the limited literature available [23], research into the potential hazards and risks associated with milk whey is imperative to establish the safety of these products.

The objective of this study was to investigate the safety aspect of ovine milk whey assessing the cytotoxicity with a viability assay. The second objective was to explore the potential role of ovine milk whey as functional ingredients in the production of foods with nutraceutical value. We examined how milk whey influences the capacity for tissue repair in a “in vitro model” of gingival fibroblast (HGF-1) cell cultures. Wound healing is a dynamic process in skin repair that encompasses various stages, including inflammation, proliferation, and the migration of different cell types, such as fibroblasts. Fibroblasts play a pivotal role in the repair process, from the later stages of inflammation to the final epithelialization of damaged tissue. In this regard, we explored the impact of ovine milk whey collected from a farm in Calabria on an in vitro model of the oral healing process obtained by mechanical injury on human gingival fibroblasts. Therefore, human gingival fibroblasts exposed to different concentrations of ovine milk whey were evaluated for cell proliferation (viability), healing capabilities, and the expression of extracellular matrix proteins. 

## 2. Materials and Methods

### 2.1. Ovine Milk Collection and Whey Preparation

Milk was gathered from an ovine dairy farm located in the province of Catanzaro (Calabria region, Italy), housing 50–70 lactating Sarda ewes at various stages of lactation. The ovine milk was collected from a bulk tank. Milk was stored at a temperature of 4 °C and subsequently transported to the laboratory at the University Magna Graecia in Catanzaro. Cream was isolated through centrifugation at 3500× *g* (at 4 °C for 30 min), and the fat content was separated and removed. Subsequently, whey was extracted from each sample using the procedure outlined in previous studies [24,25,26]. In brief, a 10% rennet solution (comprising 100% chymosin with 200 international milk-clotting units/mL, Hansen Standard Chy-Max Plus 200, Chr. Hansen, Hørshoghm, Denmark) was added until curd formation was achieved. Subsequently, orthogonal vertical cuts were made using a stainless-steel spatula to collect the milk whey. Prior to subsequent analysis, the milk whey was collected and filtered through a 0.45 µm syringe filter (Minisart, Sartorius Stedim Biotech GmbH, Göttingen, Germany). The protein content (mg/mL) of ovine milk whey was quantified using a spectrophotometer with a Quick StartTM Bradford protein assay (Bio-Rad, Hercules, CA, USA). This was performed by reading the samples in triplicate at 595 nm, and the protein amount was determined by interpolating the experimental values against standard proteins of known concentrations.

### 2.2. Cells

Human primary gingival fibroblasts were purchased from ATCC (CRL-2014) and used as a model for regenerative medicine studies since they show characteristics similar to adult stem cells. Human primary gingival fibroblasts were grown in Dulbecco’s Modified Eagle Medium (DMEM) (no. D57961; Sigma Aldrich, Milan, Italy) supplemented with 10% fetal bovine serum (no. 10270106; Life Technologies, Milan, Italy), 100 U/mL penicillin (P4333, Sigma, Milan, Italy), and 100 μmol/mL streptomycin (no. P4333; Sigma Aldrich) at 37 °C and 5% CO_2_ in a humidified atmosphere. Cells were sub-cultivated at a density of 2500 to 5000 cells per cm^2^.

### 2.3. 3-[4,5-Dimethylthiaoly]−2,5-diphenyltetrazolium Bromide (MTT) Assay

The cellular metabolic activity of human primary gingival fibroblast (HGF-1) cells exposed to increasing concentrations of ovine whey (0.01%, 0.1%, 1%, 10%) was measured by MTT assay as described in Chiarella et al. (2019) [27]. Briefly, 5 × 10^3^ cells were plated in quadruplicate on 96-well plates in 100 μL of complete growth medium with or without ovine whey. Cell viability was monitored for 72 h by adding 10% MTT solution (working concentration 0.5 mg mL) to each well every day. Cells were incubated with MTT reagent at 37 °C for 2 h and eventually isopropanol acidified with HCl; 0.08 N was added by pipetting up and down to dissolve the formazan crystals. The absorbance was determined at optical density of 595 nm with a microplate reader, Glomax (Promega, Milan, Italy). 

### 2.4. Wound Healing Assay

The scratch injury is a well-known method to investigate in vitro cell migration during wound healing. The effect of ovine milk whey on HGF-1 migration was investigated by wound healing assay according to Mesuraca et al. 2019. [28] Briefly, HGF-1 cells were seeded at a density of 5 × 10^5^ cells per well in a 6-well plate and grew until a confluent cell monolayer. Subsequently, the mechanical stretch injury was obtained by scraping the dish surface with a sterile tip (Ø = 0.1 cm). The cultures were washed twice with PBS to remove cell debris and then incubated in fresh medium without or with increasing concentrations of ovine milk whey (0.01%, 0.1%, 1%, 10%). The recolonization of the scratched region in untreated and treated HGF cells was imaged at 0 h, i.e., when the wound was produced, and at 24 and 48 h after scratching. Phase-contrast images were captured at 20× magnification under an inverted microscope (DM IL LED, Leica Microsystems Srl, Milan, Italy). Three regions per well were uniquely identified for picture acquisition. The percentage of wound coverage was determined with a plugin of ImageJ Fiji software (version 1.49).

### 2.5. Cytofluorimetric Analysis

After scratching the cell monolayer and exposing it to different concentrations of milk whey (0, 0.001, 0.01, 1, and 10%), 2 × 10^5^ cells were collected, washed with PBS, and stained with anti-CD44-PE (clone DB105, Miltenyi Biotec, Bergisch Gladbach, Germany) on ice for 30 min in the dark. Subsequently, cells were washed twice with iced PBS and acquired with BD FACSCanto II flow cytometry [29]. FlowJo software version 8.8.6 was employed for data analysis, specifically gating viable cells and quantifying the fluorescent signal as a percentage and mean fluorescent intensity.

### 2.6. Quantitative Real-Time Polymerase Chain Reaction

After 2 days of exposure, untreated cells and cells treated with increasing milk whey concentrations were rinsed with PBS, and total cellular ribonucleic acid (RNA) was extracted using TRIzol reagent (Life Technologies, Carlsbad, CA, USA) according to the manufacturer’s instructions. The RNA concentration was determined by measuring the samples’ absorbance at 260 nm using a NanoDrop 2000 spectrophotometer (Thermo Fisher Scientific, Waltham, MA, USA), and its purity was assessed with the absorbance ratios at 260/280 and 260/230 nm. For each sample, 1 μg of mRNA was reverse-transcribed into complementary DNA (cDNA) using a reverse transcriptase system kit (no. 4368814; Thermo Fisher Scientific, Milan, Italy). Quantitative real-time PCR (q-RT-PCR) was performed as previously reported [30]. Briefly, q-RT-PCR was performed using SYBR Green Universal PCR Master Mix (no. 4368706; Life Technologies). One cycle of 10 min at 95 °C was followed by 45 cycles of 10 s at 95 °C, 20 s at 58 °C, and 20 s at 72 °C. The reactions were performed in triplicate and analyzed using the DDCt method in which the relative expression of the Collagen I gene (COLI) was at first normalized by the housekeeping gene glyceraldehyde-3-phosphate dehydrogenase (GAPDH). Relative fold changes in gene expression were then determined by the 2^−ΔΔCt^ method.

Primers used in this study were COLI (Forward 5′-GTACTGGATTGACCCCAACC-3′; Reverse 5′-ACCAGACATGCCTCTTGTCC-3′) and GAPDH (Forward 5′-GGCTCTCCAGAACATCATCC-3′; Reverse 5′-TTTCTAGACGGCAGGTCAGG-3′).

### 2.7. Immunofluorescence Analysis

Immunofluorescence was conducted following the methodology described in Di Vito et al. (2023) [31]. In brief, HGF-1 cells were plated at a concentration of 4 × 10^3^ cells per cm^2^ in 12-well plates and then exposed to different concentrations of ovine milk whey (0%, 0.01%, 0.1%, 1%, and 10%) for 24 h. Cell fixation was conducted using 0.3% glutaraldehyde (G5882, Sigma, Milan, Italy) for 15 min. Subsequently, the cells were washed three times with PBS, and permeabilization was achieved with 0.1% Triton X-100 (T8787, Sigma, Milan, Italy) for 20 min. Blocking of nonspecific binding sites was achieved by incubating the cells with 1% bovine serum albumin (BSA, 05470, Sigma, Milan, Italy) for 60 min. To assess collagen deposition, both treated and untreated HGF-1 cells were incubated with anti-Collagen I antibody (catalog number ab34710; diluted at 1:500, rabbit; Abcam, Cambridge, UK) for 16 h at 4 °C, followed by incubation with Alexa Fluor 488-conjugated anti-rabbit secondary antibody (catalog number A11070; diluted at 1:400; Life Technologies) for 60 min at room temperature. Nuclei were stained with 1 μg/mL DAPI (Santa Cruz Biotechnology, Dallas, TX, USA) for 20 min at room temperature. Images were captured at 20× magnification using a Leica fluorescence microscope (Leica DM IL LED, Leica Microsystems, Milan, Italy).

### 2.8. Statistical Analysis

For statistical analyses, *t*-tests were applied with Microsoft^®^ Excel^®^ 2010 (Redmond, WA, USA); *p* < 0.05 was taken as the level of significance.

## 3. Results

### 3.1. Effect of Ovine Whey on HGF-1 Viability

An MTT assay was conducted to assess the in vitro metabolic activity of ovine whey on human primary gingival fibroblasts. HGF-1 cells were exposed to varying concentrations of ovine whey (0%, 0.01%, 0.1%, 1%, and 10%) for three days, and tetrazolium salt reduction, considered an indicator of cell viability, proliferation, and cytotoxicity, was measured every 24 h thereafter.

**Figure 1:**
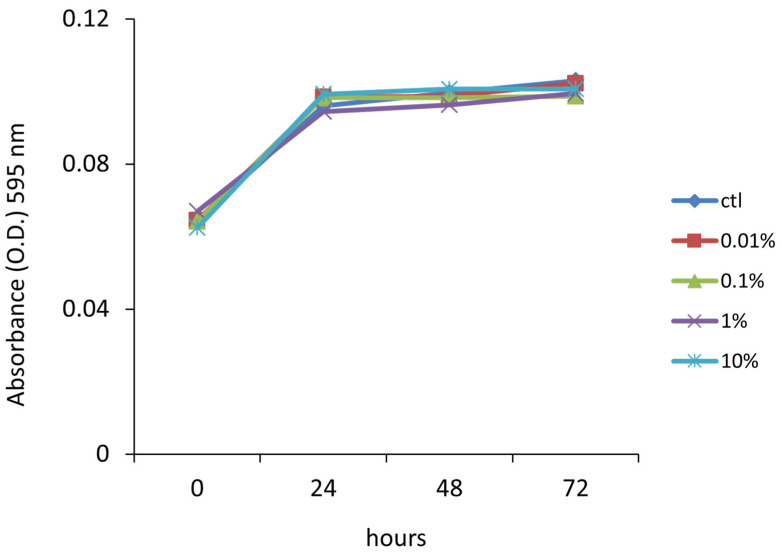
Viability of HGF-1 cells upon ovine milk whey treatment. The proliferation rate of HGF-1 cells exposed to 0.01%, 0.1%, 1%, and 10% ovine milk whey was monitored by MTT assay for 48 h. No harmful effects were observed in cells treated with varying concentrations of ovine milk whey.

No significant absorbance variations at 490 wavelengths were observed in cells incubated with 0.01%, 0.1%, 1%, and 10% ovine whey compared to control cells over time (Figure 1). The limited variation in proliferation rate can be attributed to the HGF-1 doubling time.

### 3.2. Ovine Whey Promotes Wound Repair in the In Vitro HGF-1 Gingiva Injury Model

**Figure 2:**
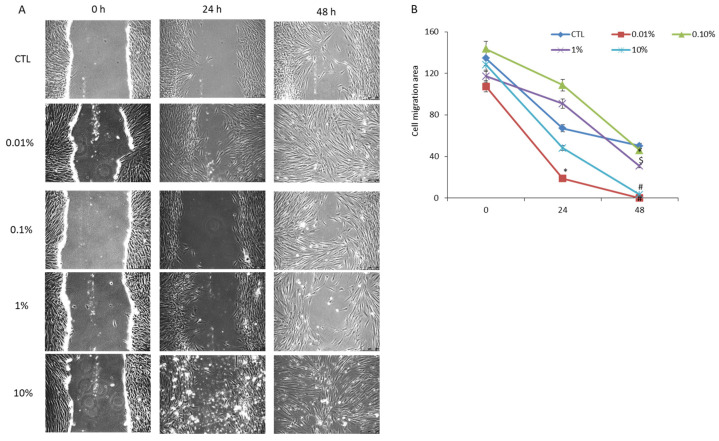
Effect of ovine milk whey on HGF-1 migratory capability. A monolayer of HGF-1 cells was scratched and treated with ovine milk whey at 0.01, 0.1, 1, and 10% for 48 h. (**A**) Cell migration was monitored microscopically by taking representative images. (**B**) The percentage of wound closure was calculated using the ImageJ software (* *p* ≤ 0.05, $ ≤0.001, # ≤0.0001).

To assess the potential of ovine whey in tissue regeneration, a wound healing assay was performed. HGF-1 cells subjected to mechanical injury through a scraping process were cultured for three days in the presence of ovine whey at concentrations of 0.01%, 0.1%, 1%, and 10%. The changes in healing rate were measured every 24 h. Interestingly, in cells treated with the lowest (0.01%) concentration of ovine whey, the percentage of wound closure was enhanced 3.5 fold compared to control cells after just 24 h. This effect was accentuated after 48 h of stimulation, at which point the wound appeared completely healed. Surprisingly, after one day, the wound area measurement was only reduced by approximately 1.2 and 1.5 fold in HGF-1 cells treated with 0.1% and 1% sheep whey, respectively, compared to the measurement taken at 0 h. However, intermediate concentrations of 0.1% and 1% sheep whey were able to induce migration in the cell culture tested within 48 h. In this case, the percentage of wound closure increased by approximately 1.11 and 1.63 times compared to that of control cells. Finally, exposure to 10% sheep whey increased wound-healing-related processes in gingival fibroblasts in a dose/time-dependent manner. Specifically, the wound area had shrunk approximately 1.5 fold after 24 h and was almost completely closed after 48 h. At that time the percentage of wound closure increased 15.6 fold compared to control. These data indicate that sheep whey has beneficial effects on the healing of gingival wounds. The image of the scratch assay is displayed in Figure 2A. All results are displayed in Figure 2B.

### 3.3. Ovine Whey Enhances CD44 Antigen Density and Promotes Collagen I Up-Regulation in the In Vitro HGF-1 Gingiva Injury Model

**Figure 3:**
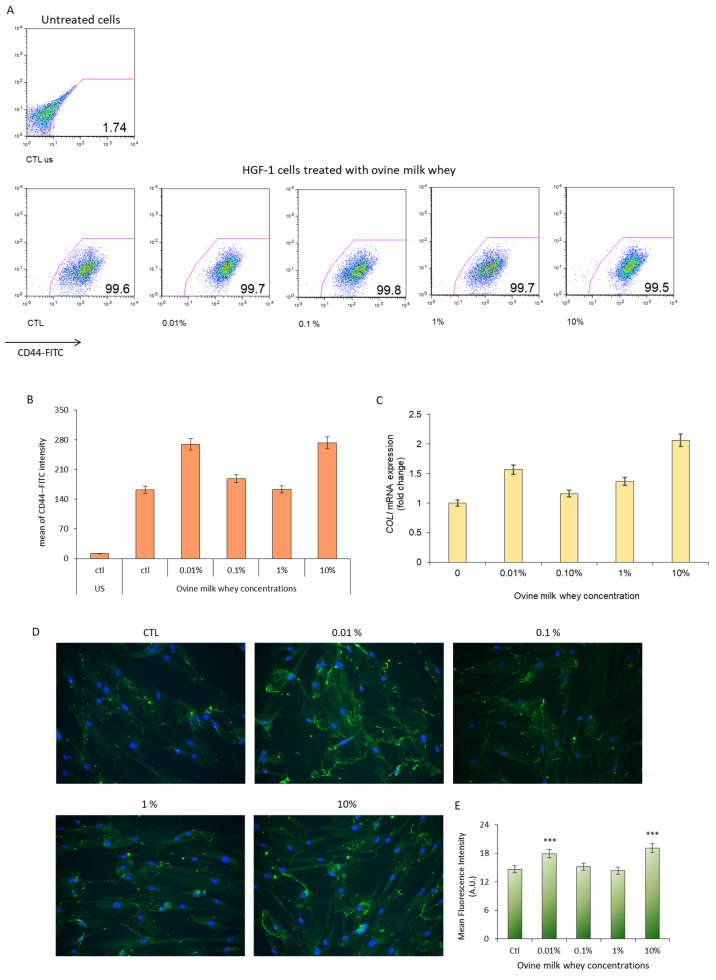
Ovine milk whey stimulates extracellular matrix deposition in HGF-1 cells. (**A**) CD44 expression was evaluated by FACS analysis in HGF-1 cells treated with 0.01%, 0.1%, 1%, and 10% ovine milk whey for 48 h. (**B**) The mean fluorescence intensity of CD44-FITC-positive cells was calculated by FlowJo software. (**C**) The mRNA levels for *COLI* were measured by q-RT-PCR in HGF-1 cells exposed to different concentration of ovine milk whey. (**D**) Representative images of Collagen I staining after treatment with ovine milk whey for 24 h. Collagen fibers, stained in green, were highly diffused in HGF-1 cells exposed to 0.01% and 10% ovine milk whey. Nuclei are stained in blue. (**E**) The mean fluorescence intensity was calculated on three images using a macro in ImageJ software. Data are presented as the mean ± standard deviation. Statistical analysis was performed using Student’s t-test in Microsoft Excel (*** *p* ≤ 0.0001).

HGF-1 cells, scratched and treated with milk whey at different concentrations, were analyzed by flow cytometry (FACS) for CD44 expression—a cell-surface glycoprotein recognized for its role in specific physiological functions, including cell proliferation, adhesion, and migration. All cell populations exhibited complete and homogeneous positivity for CD44 expression; however, the mean CD44-FITC intensity increased by approximately two-fold in cells exposed to 0.01% and 10% milk whey compared to the control. The highest (10%) and lowest (0.01%) concentrations positively influenced the distribution of fluorescence intensity (Figure 3A,B).

After 48 h of treatment with increasing ovine milk whey concentrations, COLI gene expression was determined with q-RT-PCR. The exposure to 0.01% ovine milk whey accounted for a moderate increase (+56.7%) in Collagen I gene expression with respect to untreated cells (Figure 3C). Interestingly, no significant alteration in Collagen I gene expression was reported in cells exposed to 0.1% ovine milk whey. On the other hand, a moderate (+36.6%) and very strong (+106%) up-regulation of Collagen I mRNA was reported in cells exposed to 1% and 10% ovine milk whey, respectively (Figure 3C).

The deposition of Collagen I was also investigated by immunofluorescence. Specifically, the distribution of collagen I fibers appears more evident in HGF-1 cells exposed to 0.01% and 10% ovine milk whey compared to control cells. Changes in the relative distribution of the new collagen I fibers were significantly more pronounced at the lower and higher concentrations, respectively, as determined through quantification using ImageJ software (Figure 3D,E).

## 4. Discussion

The recognition of the value of milk whey has gained considerable traction in recent decades, primarily driven by the growing demand for high-protein products [32]. This has lead to investing in new technologies useful for extracting and concentrating active substances from poor and cheap sources, such as food waste, to add them to foods [33,34,35].

Milk whey protein has found broad applications in the food industry, with numerous whey protein and peptide products showcasing multiple emerging bioactivities in both commercial markets and research laboratories. Valorization of dairy by-products into edible materials involves safety assessments. A prerequisite for any potential addition to foods is both to ensure that it does not contain harmful substances and to determine their functional effects, and few investigations have focused on these aspects. First of all, in 2008 the European Food Safety Agency (EFSA) received a request to express a scientific opinion, based upon the body of knowledge available, on the substantiation of health claims relating to whey protein [36]. Although the EFSA stated in 2010 that “No references were provided from which conclusions could be drawn for the scientific substantiation of the claimed effect” for claims such as “skeletal muscle tissue repair” and “faster recovery from muscle fatigue after exercise”, new horizons were opened based on hypotheses about the potential of whey as a “catalyst” for tissue repair processes. Until that date, however, not enough studies had been conducted on this topic. In 2017, the International Dairy Federation (IDF) partnered with Codex Alimentarius in the development, identification, elaboration, and dissemination of a science-based international standard. This standard aims to foster the clarity, composition, safety, and quality of powdered dairy permeates when used as ingredients in food [37]. Then, in 2020, the Panel of the EFSA expressed a scientific opinion on whey basic protein isolate as a novel food ingredient obtained from bovine skimmed milk, concluding that the consumption of the bovine milk whey is not nutritionally disadvantageous and is safe when the composition is known and the proposed levels of use are taken into consideration [38]. Notwithstanding the escalating efforts to reduce food waste, the progress in innovative technologies for the valorization of food by-products and the implementation of numerous laws to protect human and animal health, only a very limited number of studies have thus far tackled the safety assessment of food by-products intended for human consumption. Sattin and colleagues (2016) explored the microbial community of ricotta whey samples using NGS sequencing and underlined how pathogens can grow in pasteurized whey if it is not adequately stored and managed [39]. A study conducted by Lam and colleagues (2019) [40] has demonstrated that whey protein can enhance athletic performance without any toxic effects. Other authors have demonstrated that the administration of lactoferrin, an iron-binding glycoprotein in milk [41], does not lead to deaths or any adverse effects on the general condition of the animals. The antifungal activity of goat milk whey hydrolysate was determined against 10 toxigenic fungi from the genus Penicillium, in solid and liquid media in order to use as an ingredient for bread elaboration [42]. Following this line, even other researchers examined how to enhance milk whey by incorporating plant ingredients to create products with elevated sensory characteristics, heightened nutritional and biological values, as well as improved safety. The authors underlined how this approach conferred therapeutic, health-promoting, and functional properties to the resulting products [43]. 

In our investigation, MTT analysis was performed on the human primary gingival fibroblast to assess the effects of ovine milk whey on cell proliferation and for dose determination. Cellular metabolic activity measured by MTT assay, considered an indicator of viability, did not show any cytotoxic effect of sheep whey on gingival fibroblasts, thus reflecting the human safety profile of the ovine milk whey. However, no significant differences were found in the growth rate of cells exposed to 0.01%, 0.1%, 1%, and 10% ovine whey compared to control cells over three days. To our knowledge this investigation is the first that assayed ovine milk whey on HGF-1 cells. Other authors investigated the proliferative effect on other cell lines. In a very interesting survey, Boda and colleagues [44] performed an MTT assay on human placenta mesenchymal stem cells with milk or whey solution at increasing concentrations, alone or in combination with fetal bovine serum (FBS). The authors revealed that the cell proliferation measured through MTT at various concentrations of milk or whey alone was lower compared to the control group (FBS). They emphasized that an increase in whey concentration led to a maximum decrease in cell proliferation of 44.37% for 1% whey compared to the control (100 ± 4.39%). Although our results are not directly comparable to those of similar studies in the literature, our findings are in agreement with the authors of [45], who observed no cytotoxic activities of goat milk extract against two non-cancerous control cell lines (BJ-1and MCF-12).

The purpose of the present study was also to investigate the beneficial effect of ovine whey on an experimental in vitro model of gingivitis gingival injury. The inflammation of the gums is usually benign and resolves quickly. However, in severe cases, it could extend to the periodontium, producing periodontitis, characterized by irreversible loss of alveolar bone and attachment. Gingival fibroblasts (GFs) are vital elements that preserve the integrity and structure of the periodontal tissue. In patients with gingivitis and periodontitis, delayed gingival wound healing is frequently reported until clinical treatment restores gingival health [46,47].

Since sheep whey does not affect cell proliferation, it is possible to decouple proliferation and migration effects. Specifically, a scratch was performed on a confluent monolayer of human primary gingival fibroblasts, and then the cells were treated with ovine whey at different concentrations (0.01, 0.1, 1, and 10%) for 48 h. The outcomes showed that ovine whey has the capacity to accelerate wound healing in vitro in a dose- and time-dependent manner. Intriguingly, the HGF-1 migratory capability was still evident in the cell culture exposed to 0.01% and 10% ovine whey for 24 h (Figure 2). In these cases, the percentage of wound closure increased by 3.73-fold and 1.39-fold, respectively, compared to control cells in the same period. However, the wound was completely healed in the cells treated with 0.01% and 10% ovine whey for 48 h.

On the other hand, cells cultivated in medium supplemented with 0.1% and 1% sheep whey exhibited a different behavior. Sheep whey consistently showed positive effects, although not as pronounced as treatments with concentrations of 0.01% and 10%. In actuality, in cells treated with 0.1% and 1% sheep whey for 24 h, the migratory capabilities seemed to be delayed compared to the control, with the cell migration area being 108.68 and 94.84 versus 67.14, respectively. However, the percentage of wound closure was similar to the control in cells treated with 0.1% ovine whey for 24 h and increased by 1.6-fold in the cells exposed to 1% ovine whey for 48 h.

Interestingly, sheep whey modulates extracellular matrix remodeling by increasing the number of CD44 antigens and promoting Collagen 1 deposition in HGF-1 cells. CD44, a cell surface glycoprotein, plays a crucial role in tissue remodeling due to its ability to interact with cytoskeletal components, supporting fibroblast adhesion and migration as well as facilitating cell–cell interactions. Moreover, serving as the primary receptor for hyaluronic acid (HA), CD44 stimulates tissue remodeling and fibrosis by activating leukocytes and parenchymal cells at the site of inflammation [48]. 

Our data indicate a significant increase in the mean fluorescent intensity of CD44-stained cells in injured human primary gingival fibroblasts treated with 0.01% and 10% milk whey. A similar trend was observed in *COLI* mRNA levels as well as in the analysis of Collagen I fiber distribution by immunofluorescence (Figure 3C–E).

We found that ovine milk whey stimulates de novo production of type I collagen, contributing to the wound repair process. This phenomenon was particularly evident in HGF-1 cells exposed to 0.01% and 10% milk whey [49]. This effect appears comparable to the accumulation of collagen type I induced by other natural compounds, including propolis and curcumin or small molecules such as ML228, a known molecular activator of HIF in the in vitro wound healing model of human gingival fibroblasts [50,51,52].

In recent years, thanks to its numerous bio-components, whey protein has also attracted attention in the field of tissue repair [53]. The regenerative role of whey has been well described by several authors. Milk whey could promote proliferation and differentiation of osteoblastic cells and enhance production of collagen content in osteoblastic cells [54]. In a very interesting study performed by [55], the effects of whey protein on osteoblasts were evaluated. The authors added milk whey to the culture medium at concentrations of 0.02 and 0.1 mg/mL, and whey protein was shown to promote osteoblast growth and total osteocalcin and IGF-I content in osteoblasts in vitro. Limited research has been undertaken regarding the impact of milk whey on wound healing, a process characterized by several continuous phases involving various cells and chemical intermediates. Hemmati and colleagues (2022) investigated the potential of low-fat milk as a natural and cost-effective source for promoting the healing of full-thickness wounds. The study indicated an increase in both collagen fibers and fibroblasts and a noticeable decrease in inflammatory cells [56]. Purup and colleagues emphasized the potential use of milk protein preparations in functional foods to support gut protection and aid recovery after injury [57]. In line with our findings, the author identified a dose-dependent effect of a whey and casein hydrolysate preparation, Peptigen IF-3012. This preparation significantly increased the migration of intestinal cells by 45% at a concentration of 1 µg/mL (*p* < 0.05). However, at higher concentrations, there was a diminishing effect, and no significant impact on the migration of intestinal cells was observedInizio modulo. Another author explored the anti-inflammatory effect of milk on fibroblast and oral epithelial cells detected with PCR and immunoassays [58].

Notably, limited research has explored the safety aspects of ovine milk whey when tested on cultured cells. Our study adds to the ongoing characterization of the safety of sheep’s milk whey, with a perspective on its potential incorporation as an ingredient or utilization as a novel food. Moreover, in our study, we have demonstrated for the first time the potential of a waste dairy product in promoting tissue repair. This finding could open up exciting possibilities for re-evaluating the regenerative properties of whey. However, additional research will be necessary to assess the safety and explore the biochemical mechanisms and peptides associated with the nutraceutical potential, including on various other cell lines, to support the addition of this waste as a high-value ingredient in functional foods. As highlighted by Zhao and Ashaolu (2020) [58] in their work aimed at improving safety evaluation strategies, various approaches are suggested in addition to standard toxic and allergic examinations. These approaches include establishing the structure–activity relationship of peptides using advanced bioinformatics resources, developing sensitive detection techniques for potential toxins and allergens that might coexist in peptide products, and conducting analyses of in vivo digestion, absorption, and dietary intake for a comprehensive evaluation. Therefore, numerous further experiments and evaluations are expected in the future.

## 5. Conclusions

This work provides the effects exerted by ovine milk whey collected on a dairy farm located in the Calabria region in an in vitro model of human primary gingival fibroblast wound healing. The fascination with whey is primarily driven by its high concentrations of protein and other nitrogenous components, including bioactive peptides that offer numerous health advantages. The process of enhancing the value of dairy by-products can play a crucial role in creating novel functional food ingredients, nutraceuticals, food supplements, as well as products like fermented beverages and whey cheeses. Functional foods can be considered the result of reusing lifeless products and creating new products with beneficial effects on human health. A fundamental requirement for any potential application is that the tested sample does not pose any harm to human health. Our results suggest that ovine whey not only induces cytotoxicity, but it enhances the fibroblasts migratory capabilities in an in vitro model of gingival wounds without affecting cell viability. This effect was evident at all ovine whey concentrations tested (0.01, 0.1, 1, and 10%) and appeared particularly early at the lowest and highest concentrations of 0.01 and 10%, respectively. In addition, ovine milk whey stimulates extracellular matrix deposition, as evidenced by the increasing levels of CD44 antigen density evaluated through FACS analysis and COL1A1 expression measured via RT-Q-PCR, particularly at the concentrations of 0.01% and 10%. In conclusion, these findings suggest that ovine milk whey is safe and possess regenerative properties and thus could open exciting possibilities for re-evaluating as a valuable component in regenerative therapies for oral injuries. This important step will allow us to re-evaluate one of the main by-products of the dairy sector, with the aim of both contributing to reducing the environmental and economic impact of food waste and encouraging circular economies while respecting the world that hosts us. 

## Data Availability

The raw data supporting the conclusions of this article will be made available by the authors on request.
